# Experimental Study on the Stability of a Novel Nanocomposite-Enhanced Viscoelastic Surfactant Solution as a Fracturing Fluid under Unconventional Reservoir Stimulation

**DOI:** 10.3390/nano12050812

**Published:** 2022-02-28

**Authors:** Xiaodong Si, Mingliang Luo, Mingzhong Li, Yuben Ma, Yige Huang, Jingyang Pu

**Affiliations:** 1 School of Petroleum Engineering, China University of Petroleum (East China), Qingdao 266580, China; sixiaodong0021@163.com (X.S.); limingzhong_upc@hotmail.com (M.L.); h17667746750@163.com (Y.H.); 20200099@upc.edu.cn (J.P.); 2 Key Laboratory of Unconventional Oil and Gas Development, China University of Petroleum (East China), Ministry of Education, Qingdao 266580, China; 3 Oilfield Production Department, China Oilfield Services Limited, Tianjin 300451, China; mayb6@cnooc.com.cn

**Keywords:** nanocomposite, worm-like micelle, viscoelastic surfactant, fracturing fluid, stability

## Abstract

Fe_3_O_4_@ZnO nanocomposites (NCs) were synthesized to improve the stability of the wormlike micelle (WLM) network structure of viscoelastic surfactant (VES) fracturing fluid and were characterized by Fourier transform infrared spectrometry (FT-IR), scanning electron microscopy (SEM), energy dispersive spectrometry (EDS), X-ray diffraction (XRD) and vibrating sample magnetometry (VSM). Then, an NC-enhanced viscoelastic surfactant solution as a fracturing fluid (NC-VES) was prepared, and its properties, including settlement stability, interactions between NCs and WLMs, proppant-transporting performance and gel-breaking properties, were systematically studied. More importantly, the influences of the NC concentration, shear rate, temperature and pH level on the stability of NC-VES were systematically investigated. The experimental results show that the NC-VES with a suitable content of NCs (0.1 wt.%) shows superior stability at 95 °C or at a high shear rate. Meanwhile, the NC-VES has an acceptable wide pH stability range of 6–9. In addition, the NC-VES possesses good sand-carrying performance and gel-breaking properties, while the NCs can be easily separated and recycled by applying a magnetic field. The temperature-resistant, stable and environmentally friendly fracturing fluid opens an opportunity for the future hydraulic fracturing of unconventional reservoirs.

## 1. Introduction

In recent years, unconventional oil and gas resources, especially shale gas and tight oil, have become an important part of the world energy landscape and a substitute resource for conventional oil and gas [1,2,3,4,5]. Hydraulic fracturing technology is one of the key technologies for the efficient development of unconventional reservoirs [6,7,8]. During the fracturing operation, fracturing fluid (FF) is injected into the formation through the wellbore using a high-pressure pump, forming high well pressure, and resulting in the fracture of the reservoir rock, which opens high-conductivity fracture channels for hydrocarbons migrating from the reservoir to the wellbore [9,10]. Another function of the FF is to carry proppant (such as quartz sand) into these channels to maintain their opening [11,12]. Therefore, FF is a key component of the fracturing operation, and its performance directly affects the success of the fracturing operation. Traditional FFs are mainly water-based fracturing fluids based on polymer thickening compounds such as polyacrylamide (PAM), guar gum and their derivatives [13,14,15]. However, these polymer thickeners cannot be completely broken and degraded, and insoluble residues are preserved in the formation [16]. The residues block rock pores, reducing reservoir porosity and permeability, resulting in extremely unfavourable conditions for subsequent exploitation of oil and gas resources [17,18]. To effectively reduce damage to reservoirs, a viscoelastic surfactant (VES) fracturing fluid called clean fracturing fluid was developed [19].

The structure of VES is a worm-like micelle (WLM) network structure with hydrophilic groups facing outward and hydrophobic groups facing inward, which is self-assembled by small molecular surfactants under the action of some organic or inorganic salts (such as sodium salicylate, potassium chloride and sodium chloride) [20]. The viscoelastic surfactant solution as a fracturing fluid (VESFF) shows excellent viscoelasticity and has the advantages of good proppant-transporting performance, a strong drag reduction effect and low impact on the environment [21]. Under the action of oil, gas or formation fluids, the WLM network structure of VESFF can completely break without damaging the formation, which is ideal compared to traditional FFs [22]. However, in harsh in situ conditions of unconventional reservoirs such as high temperature and shear, the WLM network structure of VES is easily destroyed, which greatly weakens the performance of the clean FF [23,24]. Encouragingly, the rapid development and application of nanoparticles (NPs) provide new ideas to meet these challenges. Some NPs have been used to improve the structural stability of VESFF to withstand the complex environment during fracturing. Nettesheim et al. [25] found that SiO_2_ NPs incorporated into a NaNO_3_/cetyltrimethylammonium bromide (CTAB) WLM system could promote the entanglement of micelles to effectively improve the viscosity of the fracturing fluid. Similarly, Zhang et al. [26] proposed a SiO_2_ NP-enhanced CTAB/NaSal (sodium salicylate) WLM structure, while SiO_2_ NPs act as junctions of micelles to produce a cross-linked micellar system. Philippova et al. [27] further found that NP-WLM with a pseudo-crosslinking structure could increase the viscosity of the fracturing fluid by three orders of magnitude. García et al. [28] studied the influence of Al_2_O_3_ NPs on the rheological behaviour of nanoparticle-enhanced VES and found that the Al_2_O_3_ NPs increased the viscosity but not the elastic properties of the VES. In addition, some pyroelectric NPs (such as BaTiO_3_ and ZnO NPs) were investigated to improve the rheological properties and temperature resistance of VESFF, and they show good proppant-transporting performance at high temperature [29,30]. However, the abovementioned NPs do not have external response characteristics and are easily detained in the formation pores, which will damage the formation. Moreover, these NPs mixed with oil and water are difficult to separate and reuse, which not only pollutes the environment but also increases the application cost and wastes nanomaterials [31,32,33]. Fortunately, magnetic NPs can respond to an external magnetic field, which is easily separated from oil and water for reuse [34]. Magnetic NPs (such as Fe_3_O_4_ NPs) have been reported in fracture and oil/water monitoring [35,36], fracturing drag reduction [37] and enhanced oil recovery [38]. What is more, the rheological properties of magnetic NPs /WLM systems can be improved with a magnetic field [39,40]. Therefore, a nanomaterial with both magnetic and pyroelectric effects may be more suitable to improve the stability, proppant-transporting capacity, environmental protection and reusability of VESFF with NPs in complex conditions for hydraulically fracturing unconventional reservoirs.

Accordingly, this study aims to prepare a novel Fe_3_O_4_@ZnO nanocomposite-enhanced viscoelastic surfactant solution as a fracturing fluid (NC-VES) using the interactions between nanoparticles and micelles, and gain further insights into the influence and mechanism of NC concentration, shear rate, temperature and pH on the stability of the NC-VES. First, Fe_3_O_4_@ZnO nanocomposites were synthesized and characterized by Fourier transform infrared spectrometry (FT-IR), scanning electron microscopy (SEM), energy dispersive spectrometry (EDS) and vibrating sample magnetometry (VSM). Then, the NC-VES was prepared, and its properties, including settlement stability, interactions between NCs and WLMs, proppant-transporting performance and gel-breaking property, were systematically studied.

## 2. Materials and Methods

### 2.1. Materials

Ferric chloride (FeCl_3_) and ferrous sulfate heptahydrate (FeSO_4_·7H_2_O) with purities of ≥99.5 wt.% were purchased from Shanghai Aladdin Biochemical Technology Co., Ltd. (Shanghai, China). Acetoxyzinc dihydrate (Zn(Ac)_2_·2H_2_O), triethanolamine (TEOA) and sodium hydroxide (NaOH) with purities of ≥99.5 wt.% were manufactured by Shanghai Macklin Biochemical Co., Ltd. (Shanghai, China). Octadecyl trimethyl ammonium chloride (OTAC), ethyl alcohol and sodium salicylate (NaSal) with purities of ≥99 wt.% were provided by Sinopharm Chemical Reagent Co., Ltd. (Shanghai, China). The Krafft points of OTAC surfactant before and after addition of NaSal are 40 °C and 25 °C, respectively. Polyacrylamide (PAM) and guar gum were provided by PetroChina Changqing Downhole Technical Operation Company (Xi’an, China).

### 2.2. Synthesis of Fe_3_O_4_@ZnO NCs

First, Fe_3_O_4_ NPs were prepared by a chemical coprecipitation method, where 1.62 g FeCl_3_ and 2.08 g FeSO_4_·7H_2_O were dissolved in 150 mL deionized water. Then, 0.1 mol/L NaOH solution was slowly added to the mixed solution until the pH reached 10 and stirred at constant speed mixer at 300 r/min for 3 h. The Fe_3_O_4_ precipitate was then washed with ethanol and deionized water and dried at 60 °C for 10 h.

Second, ZnO NPs were synthesized by a hydrothermal method where 1.09 g Zn(Ac)_2_·2H_2_O was dissolved in 100 mL deionized water. A total of 50 mL of 0.2 mol/L NaOH solution was mixed in zinc acetate solution. Then, the mixed solution was placed in a high-pressure reactor at 160 °C with a mixing rate of 300 r/min for 8 h. The ZnO precipitate was subsequently washed with ethanol and deionized water and dried at 60 °C for 10 h.

Finally, 0.25 g Fe_3_O_4_ NPs were dispersed in 50 mL deionized water with ultrasonic vibration for 15 min. Then, 20 mL TEOA solution (1.6 mol/L) and 30 mL zinc acetate solution (0.02 mol/L) were added into the previous solution at 90 °C with stirring at 300 r/min for 10 h. Thus, the composite nanoparticles were obtained after repeated purification more than 5 times through centrifugation (Centrifuge ST16, Thermo Fisher Scientific Inc., Osterode, Lower Saxony, Germany), washing, drying and magnetic adsorption separation.

### 2.3. Preparation of the NC-VES

First, 2.87 mmol OTAC was dissolved in 80 mL deionized water at 40 °C. Fe_3_O_4_@ZnO NCs were dispersed in the OTAC solution then ultrasonically vibrated for 15 min. Then, 20 mL NaSal solution (0.156 mol/L) was added into the previous suspension solution at a mixing rate of 180 r/min for 10 min. NC-VES was obtained after standing for 12 h at room temperature. Meanwhile, VES solution with Fe_3_O_4_ nanoparticles and without nanoparticles were prepared with the same concentration (OTAC: 0.287 mmol/L; NaSal: 0.312 mmol/L) using the same methods.

### 2.4. Characterization of Nanoparticles

The main functional groups of the samples were examined by FT-IR with a Nicolet 6700 FT-IR instrument (Thermo Fisher Scientific Inc., Waltham, MA, USA). The surface morphology of the nanoparticles was observed by scanning electron microscopy (JSM-5800, JEOL Ltd., Toyoshima, Tokyo, Japan). The elemental content of the nanoparticles was examined with an energy dispersive spectrometer (JEOL Ltd., Toyoshima, Tokyo, Japan). The size of the synthesized nanoparticles was measured by a Malvern laser particle size analyzer (ZS90, Malvern Panalytical, Malvern, UK). The crystal structure of the synthesized samples was measured by X-ray diffractometer (XRD, PANalytical B.V., Almelo, The Netherlands). The magnetizing ability of the samples was evaluated through a vibrating sample magnetometer (Lake Shore 7400, Lake Shore Cryotronics Inc., Westerville, OH, USA).

### 2.5. Property Tests of the NC-VES

**Figure 1:**
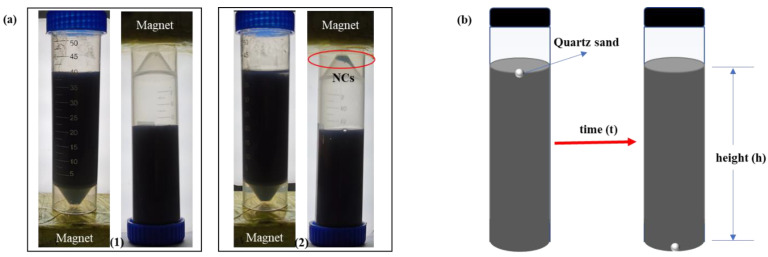
Test of the settlement stability (**a**) and proppant-transporting performance (**b**).

**Figure 2:**
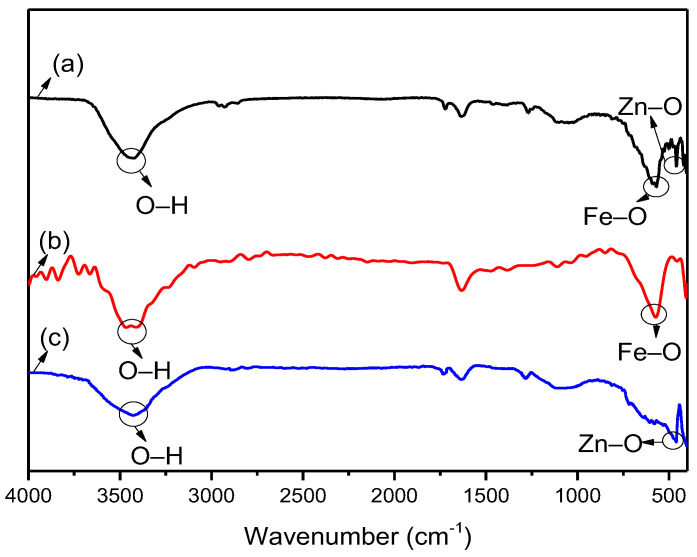
FT-IR spectra of the samples, (**a**) Fe_3_O_4_@ZnO, **(b**) Fe_3_O_4_, (**c**) ZnO.

If the network structure of the NC-VES system is unstable or seriously damaged, some or all of the NCs in the system will settle out due to the density difference between the NCs and liquid phase. In this test, the settlement rate of NCs was used to characterize the stability of the NC-VES system. The slower the settlement rate is, the better the stability of the NC-VES. The settlement rates (Equation (1)) were measured by the weighing method with a precision balance (the accuracy was 0.0001 g; Shanghai Fangrui Instrument CO., LTD., Shanghai, China). In this test, the settled NCs were separated from the liquid phase by a magnet as seen in Figure 1a-(2), while the magnet did not suck out the unsettled particles in the system as seen in Figure 1a-(1). Additionally, the effect of various factors on the settlement stability of NC-VES were studied by changing the nanoparticle concentration, shear rate, temperature and pH.

(1) $$settlement\;rate=\frac{W_{2}-W_{1}}{W_{0}}\times 100\%$$

where *W*_0_ is the total weight of the NCs added to the NC-VES, g; *W*_1_ is the weight of the empty sample bottle, g; and is the total weight of the sample bottle and settled NCs after being dried, g.

Proppant-transporting performance is one of the key properties of fracturing fluid. In the test, the settling velocity of proppant (quartz sand) in the NC-VES is used to characterize the proppant-transporting performance. As shown in Figure 1b, the settling velocity was calculated by the Formula *v* = *h*/*t*, where *h* and *t* were recorded as the vertical distance (cm) and time (s) taken for quartz sand as proppant to settle from the surface to the bottom of 40 mL NC-VES.

## 3. Results and Discussion

### 3.1. Characterization of Fe_3_O_4_@ZnO Nanocomposites

Figure 2 shows the comparison of the chemical structures of Fe_3_O_4_, ZnO and Fe_3_O_4_@ZnO based on the FT-IR spectra obtained over the wavenumber range of 400–4000 cm^−1^. The stretching vibration peak at approximately 3410 cm^−1^ in all the IR spectra is attributed to hydroxyl groups (O–H) due to the adsorbed water molecules on the surface of the samples. The peak at approximately 570 cm^−1^ represents Fe-O bonds in spectra Figure 2a,b. The stretching peak at approximately 450 cm^−1^ is related to Zn-O bonds in spectra Figure 2a,c. Both the Fe-O bond and Zn-O bond existing in the synthesized Fe_3_O_4_@ZnO composite indicate that ZnO was adsorbed on the surface of the Fe_3_O_4_ particles.

**Figure 3:**
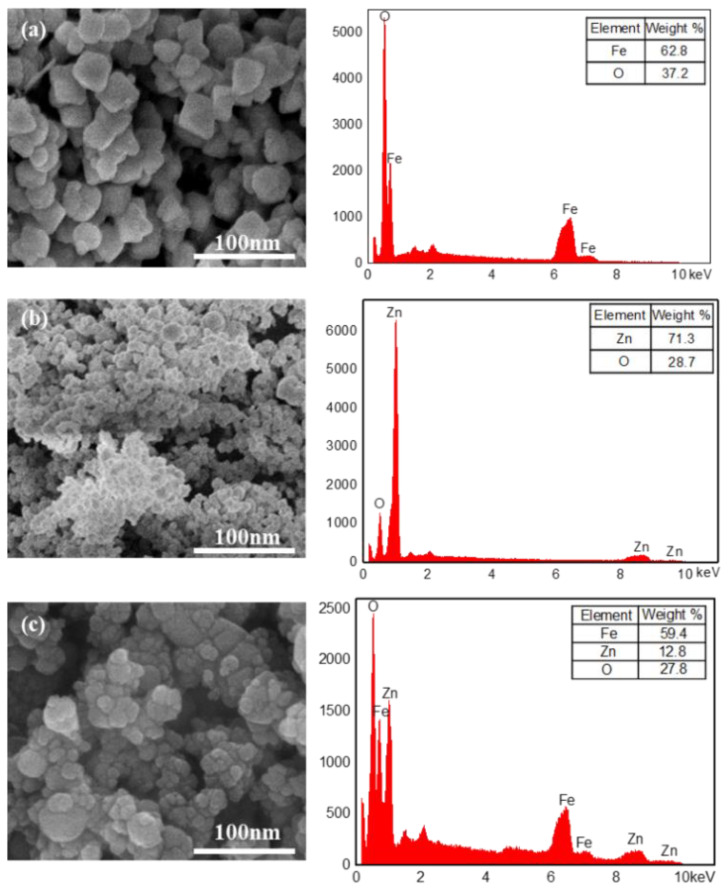
SEM (**left**) and EDS analysis (**right**) of Fe_3_O_4_ (**a**), ZnO (**b**) and Fe_3_O_4_@ZnO (**c**).

**Figure 4:**
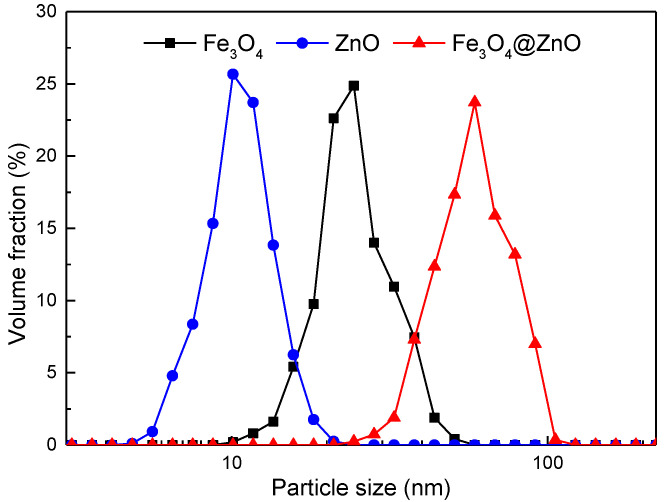
Particle size distribution of Fe_3_O_4_, ZnO and Fe_3_O_4_@ZnO nanoparticles.

The SEM images with EDS analysis of the Fe_3_O_4_ (a), ZnO (b) and Fe_3_O_4_@ZnO samples (c) are depicted in Figure 3. As seen from the SEM images, the average sizes of the synthesized Fe_3_O_4_ NPs, ZnO NPs and Fe_3_O_4_@ZnO NCs are approximately 25 nm, 10 nm and 60 nm, respectively. As shown in Figure 4, the medium diameters of the three nanoparticles with a narrow size distribution, were consistent with the observation results of the SEM images. The pure Fe_3_O_4_ NPs appear to exhibit an octahedral structure (Figure 3a), while the ZnO NPs display a spherical-like structure with smaller dimensions (Figure 3b). Figure 3c clearly shows that ZnO NPs are successfully adsorbed on the surface of Fe_3_O_4_ NPs. Additionally, the comparison results of the EDS analysis further showed that the composite nanoparticles were successfully synthesized. Meanwhile, the mass ratio of Fe_3_O_4_ and ZnO (5.14:1) in the Fe_3_O_4_@ZnO NCs was obtained from the EDS analysis. Besides, in all EDS diagrams, the oxygen content higher than the theoretical value was mainly attributed to the adsorption of water molecules.

**Figure 5:**
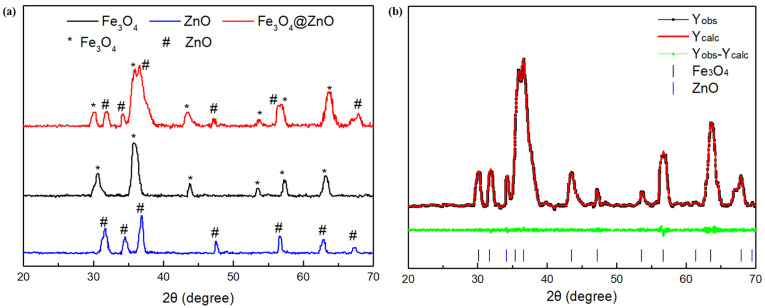
XRD patterns for synthesized nanoparticles (**a**) and Rietveld refinement of the XRD data of the Fe_3_O_4_@ZnO composite (**b**).

XRD analysis of synthesized Fe_3_O_4_, ZnO and Fe_3_O_4_@ZnO nanoparticles and Rietveld refinement of the XRD data of the Fe_3_O_4_@ZnO composite were shown in Figure 5. As shown in Figure 5a, the characteristic peaks and Bragg lattice planes reported at 30.08° (220), 35.42° (311), 43.08° (400), 53.56° (422), 56.98° (511) and 62.63° (440) are related to Fe_3_O_4_ structure, while the characteristic peaks and Bragg lattice planes reported at 31.72° (100), 34.44° (002), 36.21° (101), 47.49° (102), 56.51° (110), 62.81° (103) and 67.86° (112) are related to ZnO structure, which are in accordance with the patterns of standard pure Fe_3_O_4_ and ZnO, respectively [41,42]. Almost no obvious impurity peak was observed, which confirmed the high purity for the synthesized samples. As shown in Figure 5b, Rietveld refinement with good fit (goodness of fit *x*^2^ = 1.19) was carried out to obtain more crystal phase information of the composite. Parameters for Fe_3_O_4_ (crystal structure: cubic; lattice constant: a = 8.372 Å; space group: Fd$\bar{3}$m) and parameters for ZnO (lattice constant: a = 3.249 Å, c = 5.208 Å; crystal structure: hexagonal; space group: P63mc) were obtained. Meanwhile, the mass ratio of Fe_3_O_4_ and ZnO (4.91:1) in the Fe_3_O_4_@ZnO NCs from the Rietveld refinement is consistent with the EDS test within the range of allowable error. The results revealed that Fe_3_O_4_@ZnO NCs with a high purity were successfully synthesized.

**Figure 6:**
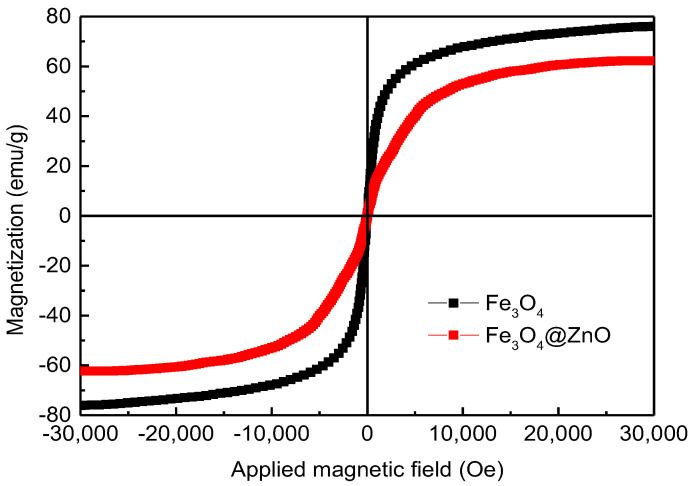
Magnetization of the Fe_3_O_4_ NPs and Fe_3_O_4_@ZnO NCs.

Figure 6 shows the magnetization of the Fe_3_O_4_ and Fe_3_O_4_@ZnO samples. It is clearly observed that the magnetization curves for the measured samples are S-shaped. When the magnetic field is zero, the remanence and coercive force are close to zero, indicating that the synthesized Fe_3_O_4_ NPs and Fe_3_O_4_@ZnO NCs possess good soft magnetic and practically superparamagnetic properties. The saturation magnetization measured for pure Fe_3_O_4_ NPs is 76.03 emu/g, while the saturation magnetization of Fe_3_O_4_@ZnO NCs decreases slightly but still maintains a high value of 62.26 emu/g. The high magnetization ensures that the Fe_3_O_4_@ZnO NCs have strong magnetic response ability and are easily separated from the liquid phase by magnetic field during quantitatively measuring the settlement rate of NCs (seen in Section 2.5). On the other hand, Fe_3_O_4_@ZnO NCs is simply recycled from the dispersed solution by using a magnet (seen in Section 3.3). What is more, the mass ratio of Fe_3_O_4_ and ZnO calculated from the comparative saturation magnetization is 5.02:1, consistent with the above tests (EDS analysis and XRD analysis), which further proves the purity of the synthetic Fe_3_O_4_@ZnO NCs.

### 3.2. Stability of the NC-VES

#### 3.2.1. Effects of Shear Rate and NC Concentration

**Figure 7:**
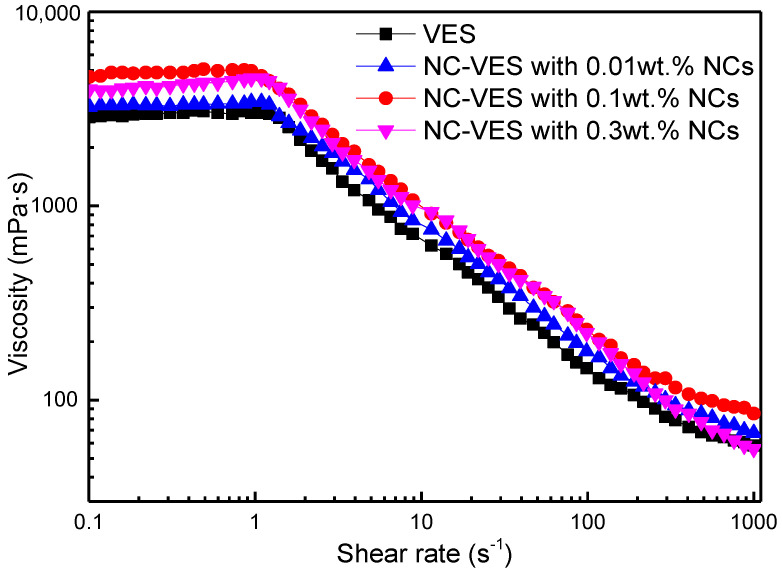
Viscosity as a function of shear rate at different NC concentrations.

The shear rate-dependent viscosities of the VESFF for different NC concentrations was studied with shear rates ranging from 0.1 to 1000 s^−1^ at 25 °C (tested with an Anton Paar rheometer, Physica MCR 302, Anton Paar GmbH, Graz, Austria). Figure 7 shows that the viscosity remains unchanged at low shear rates, while a notably reduced slope is observed under high shear rates, and the shear-thinning phenomenon occurs in all samples. The dependence of viscosity on shear rate is usually explained by shear banding behaviour, which has been reported in previous research results [43,44,45,46]. It is found that the NCs can improve the viscosity of the VES system. When the concentration of the NCs is low (0.01 wt.%), the viscosity increases but is not clear compared with the VES without NCs. When the NC concentration reaches 0.1 wt.%, the viscosity at 170 s^−1^ of the NC-VES is higher than VES without NCs. However, when the NC concentration is higher (0.3 wt.%), the viscosity of NC-VES decreases sharply at high shear rates.

**Figure 8:**
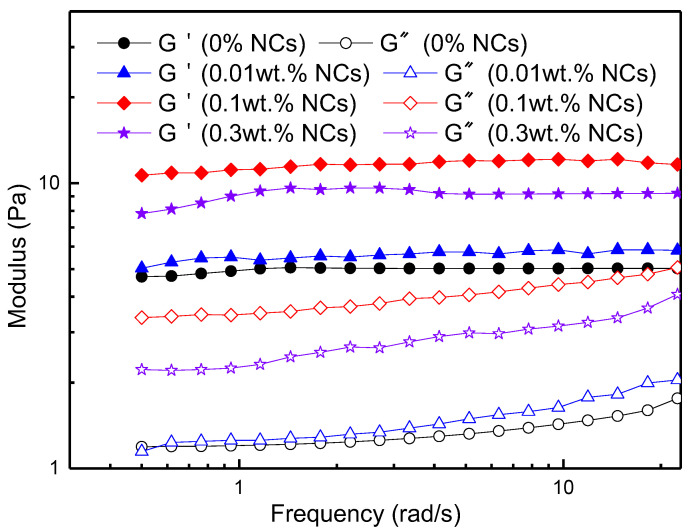
Variations of storage modulus (G′) and loss modulus (G″) with frequency at 25 °C.

Dynamic modulus (storage modulus G′ and loss modulus G″) as a function of frequency is shown in Figure 8. Within the measured test frequency range, the storage modulus remains almost unchanged, while the loss modulus increases gradually. For NC-VES systems, the storage modulus and loss modulus are greater than those of the VES system, suggesting that the WLM network in VES is strengthened by NCs. Here, the nanoparticles are incorporated into worm-like micellar systems to form nanoparticle-micelle junctions as the connection point of WLMs, which improves the structural stability and viscoelasticity of the WLM systems [47,48,49]. Combined with Figure 7 and Figure 8, this indicates that the NC concentration (0.1 wt.%) enables the NC-VES system to possess sufficient stability and high viscosity, maintaining good proppant-transporting performance during fracturing operations. Therefore, subsequent research on the influencing factors of fracturing fluid focuses on the NC-VES system with a 0.1 wt.% NC concentration. 

#### 3.2.2. Effect of Temperature

**Figure 9:**
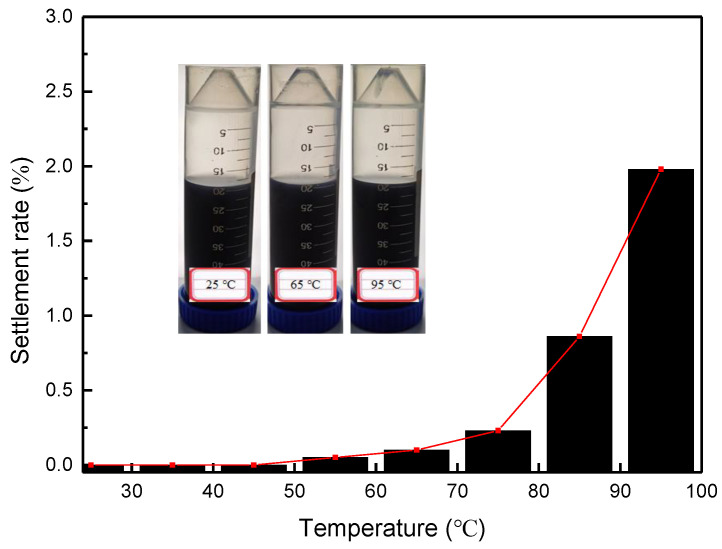
Settlement rate as a function of temperature, in which the insertions from left to right refer to the stable state of the NC-WLM system at 25 °C, 65 °C and 95 °C respectively.(OTAC: 0.287 mmol/L; NaSal: 0.312 mmol/L).

All samples were heated to the corresponding experimental temperature by a water bath and held for 10 h, then the settlement rate of NCs in each sample was obtained by weighing and calculation, as shown in Figure 9. The results show that when the temperature increased from 25 to 65 °C, the settlement rate of NCs increased from 0 to 0.1%, while almost no sedimentation phenomenon occurred, indicating that the NC-WLM network structure of the system was very stable in the low to medium temperature range. As the temperature continued to rise, the sedimentation rate increased slightly, but even at a high temperature of 95 °C, the settlement rate was only about 2%, indicating that the temperature resistance of the system was very good. Conventional VESFF is a WLM network structure formed by the spontaneous aggregation and self-assembly of high-concentration surfactant molecules, which has a specific viscoelasticity and can meet the needs of fracturing in medium- and low–temperature reservoirs. However, the WLM structure in traditional VESFF is easily broken at high temperatures, because the interaction between molecules such as van der Waals forces and hydrogen bonding forces weakens and the stability of aggregates decreases [23], leading to a sharp decrease in the viscosity of the system, which severely weakens the performance of the fracturing fluid.

**Figure 10:**
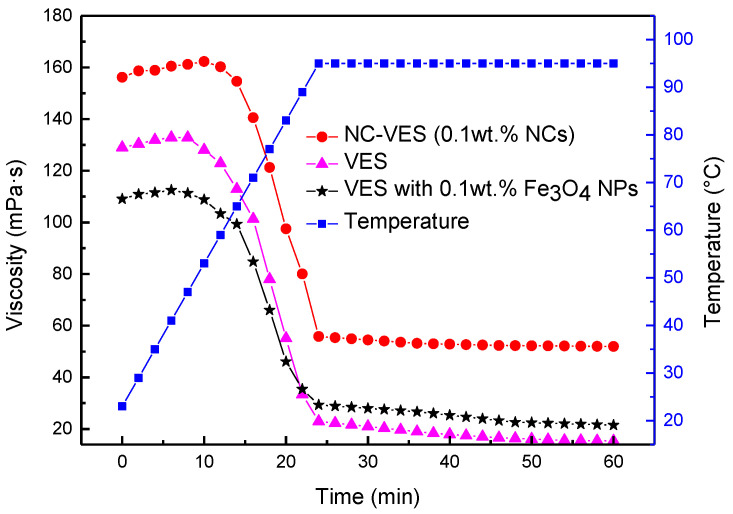
Viscosity as a function of time at high temperature (heated to 95 °C).

**Figure 11:**
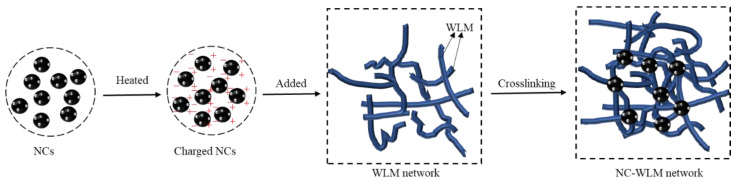
Schematic diagram of the stability of the NC-WLM network structure at high temperatures.

The viscosities as a function of time at 95 °C with a constant heating rate of 3 °C/min and a shear rate of 170 s^−1^ are displayed in Figure 10. When the temperature is lower than 60 °C, a slight increase in viscosity was observed in all three curves. However, as the temperature continues to increase to 95 °C, the viscosity of the samples decreases sharply. The viscosity of VES with Fe_3_O_4_ NPs is lower than that without NPs at 95 °C. The reason is that a certain amount of surfactant molecules are adsorbed on the surface of Fe_3_O_4_ NPs, resulting in a decrease in the surfactant concentration involved in WLM structure in the liquid phase [50]. Compared with the other two systems, the high-temperature resistance of the NC-VES system is mainly attributed to the ZnO NPs on the surface of the NCs. As shown in Figure 11, the ZnO nanoparticles adsorbed on the surface of NCs possess a pyroelectric effect, releasing charges with increasing temperature. The charged NCs easily adsorb micelles and play a role in the junction of the WLM network structure. The electrostatic screening of charged WLMs promotes further growth of wormlike micelles, and maintains good stability of the WLM network structure [29]. Therefore, the NCs incorporated into the WLM network act as a skeleton-like structure, greatly improving the stability of the NC-WLM system at high temperature.

#### 3.2.3. Effect of pH

**Figure 12:**
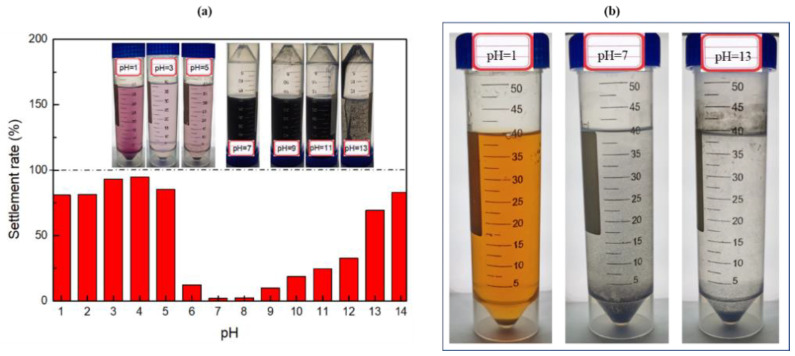
Settlement rate as a function of pH (**a**) and behaviour of NCs in deionized water without of WLMs (**b**).

As shown in Figure 12a, in a highly acidic environment (pH = 1–5), the settlement phenomenon of the system is very clear, while the settlement rate is as high as 94.9%. This shows that the system cannot exist stably in strong acids, which can be attributed to two aspects. On the one hand, in the presence of a strong acid, some NCs react with H^+^ (Fe_3_O_4_ + 8H^+^ = Fe^2+^ + 2Fe^3+^ + 4H_2_O, ZnO + 2H^+^ = Zn^2+^ + H_2_O) and are dissolved in the solution. The solution will then exhibit the crimson colour of the Fe^2+^ and Fe^3+^ aqueous solutions. On the other hand, excessive H^+^ leads to an increase in the repulsive force between the head groups of the OTAC molecule and destroys the WLM network structure, which results in large settlement of NCs [51,52]. From the results, when pH = 1, the settlement rate decreases, which does not mean that the settlement stability of the NC-VES becomes better, but that more NCs have been dissolved in the solution. Therefore, it can be considered that the stronger the acidity, the worse the settlement stability of the system. When the pH is weakly acidic–neutral–weakly alkaline (pH = 6–9), the settlement rate of the system is very small. Meanwhile, Figure 12b shows that it is difficult for NCs to form stable suspension in water without WLMs even under neutral conditions, because the particle density (about 5.26 g/cm^3^) is much greater than that of the liquid. In the strongly alkaline environment (pH = 10–14), the settlement rate of NCs increases rapidly with increasing pH. When pH ≥ 13, a large number of NCs settle out. On the one hand, ZnO is dissolved by alkaline solution (ZnO + 2OH^−^ + H_2_O = [Zn(OH)_4_]^2−^). On the other hand, an excessive OH^−^ destroys the self-assembly mechanism of surfactant molecules, leading to micelles unsuccessful for connecting with each other and assembling into a WLM network structure [53]. Therefore, the pH environment has a strong impact on the stability of the NC-VES, with the system being able to maintain a good stability in a wide range of pH = 6–9.

### 3.3. Proppant-Transporting Performance and Gel-Breaking Property

**Figure 13:**
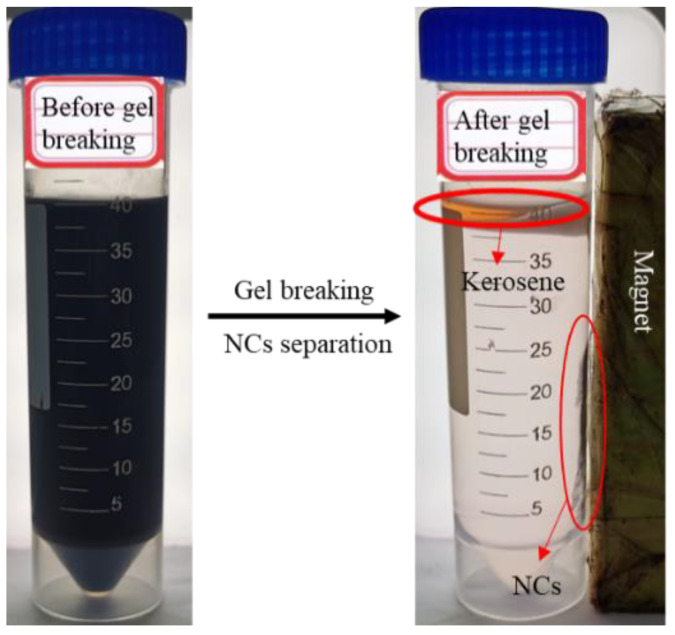
Gel-breaking test and the separation of NCs by a magnet.

In the process of a hydraulic fracturing operation, fracturing fluid is pumped into the formation, cracking the rock formation and forming a fracture channel to improve the oil and gas production efficiency. To prevent fracture closure, proppants (such as quartz sand) are transported by fracturing fluid into the formation to prop fractures, keeping the fracture open for a long time under formation pressure. Therefore, it is very important that the fracturing fluid possess good proppant-transporting performance, which is one of the key factors for the success of fracturing operations. In this work, the proppant-transporting performance of four fracturing fluids was compared, and the results are shown in Table 1. Compared with conventional fracturing fluids (Guar FF and PAM FF), the VES systems (NC-VES and VESFF) show better proppant-transporting performance. The settlement velocity of quartz sand in the NC-VES is only 0.52 × 10^−3^ cm/s, far less than 0.08 cm/s, which displays superior proppant-transporting performance [54]. In addition, by adding a small amount of kerosene to NC-VES, gel breaking occurs rapidly with no residue, and the NCs can be easily recycled using a magnet to apply a magnetic field, as shown in Figure 13. The results imply that the NC-VES system is a low-damage, environmentally friendly and cost-saving fracturing fluid.

## 4. Conclusions

In this study, the influence and mechanism of NC concentration, shear rate, temperature and pH on the settlement stability of the NC-VES fracturing fluid was systematically investigated. First, NCs with good magnetic response ability were synthesized and acted as junctions of micelles to improve the stability of the WLM network structure. The results showed that the NC-VES system with the optimal concentration of 0.1 wt.% possesses good shear and temperature resistance. At high temperatures (such as 95 °C), the ZnO NPs with a pyroelectric effect on the surface of NCs can effectively reduce the decomposition of the WLM network structure to prevent the NCs from settling out of the fracturing fluid. Strong acid and strong alkaline solutions seriously damage the NC-VES network structure by dissolving NCs and hindering the self-assembly behaviour of surfactant molecules, resulting in an acceptable pH range of 6~9. In addition, the settling velocity of quartz sand in the system at room temperature was only 0.52 × 10^−3^ cm/s, indicating that the NC-VES has good proppant-transporting performance. Finally, no residue was found after gel breaking of the NC-VES and the recovery of NCs by magnetic adsorption, implying no damage to the formation and the environment. A new type of temperature-resistant, stable and environmentally friendly fracturing fluid was thus provided for hydraulic fracturing of unconventional reservoirs.

## Figures and Tables

**Table 1:** Settlement velocity of different FF samples at room temperature.

Samples	Constituents (per 100 mL Water)	Viscosity (mPa·s)	Settlement Velocity (cm/s)
NC-VES	1 wt.% OTAC + 0.5 wt.% NaSal + 0.1 wt.% NCs	158	0.52 × 10^−3^
VESFF	1 wt.% OTAC + 0.5 wt.% NaSal	130	7.29 × 10^−3^
Guar FF	0.35 wt.% Guar	51	0.18
PAM FF	0.35 wt.% PAM	59	0.11

## Data Availability

Data are contained within the article.

## References

[1] Luo M.L., Si X.D., Zhang Y., Yuan Z.H., Yang D.Y., Gong J. (2017). Performance evaluation of water control with nanoemulsion as pre-pad fluid in hydraulically fracturing tight gas formations. Energy Fuels.

[2] Andrés J.C., Omar J.G., Lazaros G.P., Gintaras V.R. (2018). Disclosing water-energy-economics nexus in shale gas development. Appl. Energy.

[3] Tiffany L., Doug D., Shinji M. (2019). Comparison of the degree of fouling at various flux rates and modes of operation using forward osmosis for remediation of produced water from unconventional oil and gas development. Sci. Total Environ..

[4] Luo M.L., Jia X.H., Si X.D., Luo S., Zhan Y.P. (2021). A novel polymer encapsulated silica nanoparticles for water control in development of fossil hydrogen energy-tight carbonate oil reservoir by acid fracturing. Int. J. Hydrogen Energy.

[5] Apergis N., Mustafa G., Dastidar S.G. (2021). An analysis of the impact of unconventional oil and gas activities on public health: New evidence across Oklahoma counties. Energy Econ..

[6] Vishkai M., Gates I. (2019). On multistage hydraulic fracturing in tight gas reservoirs: Montney Formation, Alberta, Canada. J. Pet. Sci. Eng..

[7] Li Y.Y., Hu W., Zhang Z.H., Zhang Z.B., Shang Y.J., Han L.L., Wei S.Y. (2021). Numerical simulation of hydraulic fracturing process in a naturally fractured reservoir based on a discrete fracture network model. J. Struct. Geol..

[8] Liu J.R., Sheng J.J., Emadibaladehi H., Tu J.W. (2021). Experimental study of the stimulating mechanism of shut-in after hydraulic fracturing in unconventional oil reservoirs. Fuel.

[9] Javadpour F., McClure M., Naraghi M.E. (2015). Slip-corrected liquid permeability and its effect on hydraulic fracturing and fluid loss in shale. Fuel.

[10] Etoughe P., Siddhamshetty P., Cao K.Y. (2018). Incorporation of sustainability in process control of hydraulic fracturing in unconventional reservoirs. Chem. Eng. Res. Des..

[11] Zhao X., Guo J.C., Peng H., Pan R., Aliu A.O., Lu Q.L., Yang J. (2017). Synthesis and evaluation of a novel clean hydraulic fracturing fluid based on star-dendritic polymer. J. Nat. Gas Sci. Eng..

[12] Xu T., Mao J.C., Zhang Y.Z., Yang X.J., Lin C., Du A.Q., Zhang H. (2021). Application of gemini viscoelastic surfactant with high salt in brine-based fracturing fluid. Colloids Surf. A.

[13] Baruah A., Pathak A.K., Ojha K. (2015). Phase behaviour and thermodynamic properties of lamellar liquid crystal developed for viscoelastic surfactant based fracturing fluid. Chem. Eng. Sci..

[14] Chauhan G., Verma A., Hazarika A., Ojha K. (2017). Rheological, structural and morphological studies of Gum Tragacanth and its inorganic SiO_2_ nanocomposite for fracturing fluid application. J. Taiwan Inst. Chem. Eng..

[15] Yang B., Zhao J.Z., Mao J.C., Tan H.Z., Zhang Y., Song Z.F. (2019). Review of friction reducers used in slickwater fracturing fluids for shale gas reservoirs. J. Nat. Gas Sci. Eng..

[16] Wang J., Holditch S.A., McVay D.A. (2012). Effect of gel damage on fracture fluid cleanup and long-term recovery in tight gas reservoirs. J. Nat. Gas Sci. Eng..

[17] Liang X.Y., Zhou F.J., Liang T.B., Wang C.Z., Wang J., Yuan S. (2020). Impacts of low harm fracturing fluid on fossil hydrogen energy production in tight reservoirs. Int. J. Hydrogen Energy.

[18] Yan Z.H., Dai C.L., Zhao M.W., Sun Y.P., Zhao G. (2016). Development, formation mechanism and performance evaluation of a reusable viscoelastic surfactant solution as fracturing fluid. J. Ind. Eng. Chem..

[19] Samuel M.M., Card R.J., Nelson E.B., Brown J.E., Vinod P.S., Temple H.L., Qu Q., Fu D.K. (1999). Polymer-free fluid for fracturing applications. SPE Drill. Complet..

[20] García B.F., Saraji S. (2019). Mixed in-situ rheology of viscoelastic surfactant solutions using a hyperbolic geometry. J. Non-Newton. Fluid Mech..

[21] Shibaev A.V., Aleshina A.L., Arkharova N.A., Orekhov A.S., Kuklin A.I., Philippova O.E. (2020). Disruption of cationic/anionic viscoelastic surfactant micellar networks by hydrocarbon as a basis of enhanced fracturing fluids clean-up. Nanomaterials.

[22] Mushi S.J., Kang W.L., Yang H.B., Wang P.X., Hou X.Y. (2020). Viscoelasticity and microstructural properties of zwitterionic surfactant induced by hydroxybenzoate salt for fracturing. J. Mol. Liq..

[23] Wu X.P., Song Z.H., Zhen J.W., Wang H.B., Yao L.S., Zhao M.W., Dai C.L. (2020). A smart recyclable VES fluid for high temperature and high pressure fracturing. J. Pet. Sci. Eng..

[24] Wu X.P., Zhang Y., Sun X., Huang Y.P., Dai C.L., Zhao M.W. (2018). A novel CO_2_ and pressure responsive viscoelastic surfactant fluid for fracturing. Fuel.

[25] Nettesheim F., Liberatore M.W., Hodgdon T.K. (2008). Influence of nanoparticle addition on the properties of wormlike micellar solutions. Langmuir.

[26] Zhang Y., Dai C.L., Qian Y., Fan X.Q., Wu Y.N., Wu X.P. (2018). Rheological properties and formation dynamic filtration damage evaluation of a novel nanoparticle-enhanced VES fracturing system constructed with wormlike micelles. Colloids Surf. A.

[27] Philippova O.E., Molchanov V.S. (2019). Enhanced rheological properties and performance of viscoelastic surfactant fluids with embedded nanoparticles. Curr. Opin. Colloid Interface Sci..

[28] García B.F., Saraji S. (2020). Linear rheology of nanoparticle-enhanced viscoelastic surfactants. J. Mol. Liq..

[29] Luo M.L., Jia Z.L., Sun H. (2012). Rheological behavior and microstructure of an anionic surfactant micelle solution with pyroelectric nanoparticle. Colloids Surf. A.

[30] Baruah A., Khilendra K., Pathak A.K., Ojha K. (2016). Study on rheology and thermal stability of mixed (nonionic–anionic) surfactant based fracturing fluids. AICHE J..

[31] Díez R., Medina O.E., Giraldo L.J., Cortés F.B., Franco C.A. (2020). Development of nanofluids for the inhibition of formation damage caused by fines migration: Effect of the interaction of quaternary amine (CTAB) and MgO nanoparticle. Nanomaterials.

[32] Franco C.A., RichardZabala R., Cortés F.B. (2017). Nanotechnology applied to the enhancement of oil and gas productivity and recovery of Colombian fields. J. Pet. Sci. Eng..

[33] Giraldo L.J., Diez R., Acevedo S., Cortés F.B., Franco C.A. (2021). The effects of chemical composition of fines and nanoparticles on inhibition of formation damage caused by fines migration: Insights through a simplex-centroid mixture design of experiments. J. Pet. Sci. Eng..

[34] Dong P., Chen X., Guo M.T., Wu Z.Y. (2021). Heterogeneous electro-Fenton catalysis with self-supporting CFP@MnO_2_-Fe_3_O_4_/C cathode for shale gas fracturing flowback wastewater. J. Hazard. Mater..

[35] Sengupta S. An innovative approach to image fracture dimensions by injecting ferrofluids. Proceedings of the Abu Dhabi International Petroleum Conference and Exhibition.

[36] Rahmani A.R., Bryant S.L., Huh C. (2015). Crosswell magnetic sensing of superparamagnetic nanoparticles for subsurface applica-tions. SPE J..

[37] Luo M.L., Si X.D., Li M.Z., Jia X.H., Yang Y.L., Zhan Y.P. (2021). Experimental study on the drag reduction performance of clear fracturing fluid using wormlike surfactant micelles and magnetic nanoparticles under a magnetic field. Nanomaterials.

[38] Ali A.M., Yahya N., Qureshi S. (2020). Interactions of ferro-nanoparticles (hematite and magnetite) with reservoir sandstone: Implications for surface adsorption and interfacial tension reduction. Pet. Sci..

[39] Molchanov V.S., Pletneva V.A., Klepikov I.A., Razumovskaya I.V., Philippova O.E. (2018). Soft magnetic nanocomposites based on adaptive matrix of wormlike surfactant micelles. RSC Adv..

[40] Claracq J., Sarrazin J., Montfort J.P. (2004). Viscoelastic properties of magnetorheological fluids. Rheol. Acta.

[41] Christus A., Ravikumar A., Panneerselvam P., Radhakrishnan K. (2018). A novel Hg(II) sensor based on Fe_3_O_4_@ZnO nanocomposite as peroxidase mimics. Appl. Surf. Sci..

[42] Nadafan M., Sabbaghan M., Sofalgar P., Anvari J.Z. (2020). Comparative study of the third-order nonlinear optical properties of ZnO/Fe_3_O_4_ nanocomposites synthesized with or without Ionic Liquid. Opt. Laser Technol..

[43] Croce V., Cosgrove T., Dreiss C.A., King S., Maitland G., Hughes T. (2005). Giant micellar worms under shear:  A rheological study using SANS. Langmuir.

[44] Chu Z.L., Feng Y.J., Su X., Han Y.X. (2010). Wormlike micelles and solution properties of a C22-tailed amidosulfobetaine surfactant. Langmuir.

[45] Gao Z.B., Dai C.L., Sun X., Huang Y.P., Gao M.W., Zhao M.W. (2019). Investigation of cellulose nanofiber enhanced viscoelastic fracturing fluid system: Increasing viscoelasticity and reducing filtration. Colloids Surf. A.

[46] Yin H.Y., Feng Y.J., Li P.X., Doutch J., Han Y.X., Mei Y.J. (2019). Cryogenic viscoelastic surfactant fluids: Fabrication and application in a subzero environment. J. Colloid Interface Sci..

[47] Fanzatovich I.I., Aleksandrovich K.D., Rinatovich I.A. (2016). Supramolecular system based on cylindrical micelles of anionic surfactant and silica nanoparticles. Colloids Surf. A.

[48] Zhu J.Y., Yang Z.Z., Li X.G., Hou L.L., Xie S.Y. (2019). Experimental study on the microscopic characteristics of foams stabilized by viscoelastic surfactant and nanoparticles. Colloids Surf. A.

[49] Zhang Y., Dai C.L., Wu X.P., Wu Y.N., Li Y.Y., Huang Y.P. (2019). The construction of anhydride-modified silica nanoparticles (AMSNPs) strengthened wormlike micelles based on strong electrostatic and hydrogen bonding interactions. J. Mol. Liq..

[50] Helgeson M.E., Hodgdon T.K., Kaler E.W., Wagner N.J., Vethamuthu M., Ananthapadmanabhan K.P. (2010). Formation and rheology of viscoelastic “double networks” in wormlike micelle−nanoparticle mixtures. Langmuir.

[51] Jiao W.X., Wang Z., Liu T.Q., Li X.F., Dong J.F. (2021). pH and light dual stimuli-responsive wormlike micelles with a novel Gemini surfactant. Colloids Surf. A.

[52] Fu H.R., Duan W.M., Zhang T.L., Xu K., Zhao H.F., Yang L., Zheng C.C. (2021). Preparation and mechanism of pH and temperature stimulus-responsive wormlike micelles. Colloids Surf. A.

[53] Liu F., Liu D.J., Zhou W.J., Wang S., Chen F., Wei J.J. (2020). Weakening or losing of surfactant drag reduction ability: A coarse-grained molecular dynamics study. Chem. Eng. Sci..

[54] Wu H.R., Zhou Q., Xu D.R., Sun R.X., Wang P.Y. (2018). SiO_2_ nanoparticle-assisted low-concentration viscoelastic cationic surfactant fracturing fluid. J. Mol. Liq..

